# Central serotonin modulates neural responses to virtual violent actions in emotion regulation networks

**DOI:** 10.1007/s00429-018-1693-2

**Published:** 2018-06-08

**Authors:** Dhana Wolf, Martin Klasen, Patrick Eisner, Florian D. Zepf, Mikhail Zvyagintsev, Nicola Palomero-Gallagher, René Weber, Albrecht Eisert, Klaus Mathiak

**Affiliations:** 1 Department of Psychiatry, Psychotherapy, and Psychosomatics, Medical Faculty, RWTH Aachen, Pauwelsstraße 30, 52074 Aachen, Germany; 2 Centre and Discipline of Child and Adolescent Psychiatry, Psychosomatics and Psychotherapy, Division of Psychiatry and Clinical Neurosciences and Division of Paediatrics and Child Health, School of Medicine, The University of Western Australia, Perth, Australia; 3 Specialised Child and Adolescent Mental Health Services, Department of Health in Western Australia, Perth, Australia; 4 Institute of Neuroscience and Medicine (INM-1), Research Centre Jülich, Jülich, Germany; 5 Media Neuroscience Lab, Department of Communication, University of California Santa Barbara, Santa Barbara, CA USA; 6 Department of Pharmacy, RWTH Aachen, Aachen, Germany; 7 Department of Pharmacology and Toxicology, RWTH Aachen, Aachen, Germany; 8 JARA-Translational Brain Medicine, Aachen, Germany

**Keywords:** SSRI, Serotonin, Virtual violence, Medial prefrontal cortex, Pharmaco-fMRI

## Abstract

Disruptions in the cortico-limbic emotion regulation networks have been linked to depression, anxiety, impulsivity, and aggression. Altered transmission of the central nervous serotonin (5-HT) contributes to dysfunctions in the cognitive control of emotions. To date, studies relating to pharmaco-fMRI challenging of the 5-HT system have focused on emotion processing for facial expressions. We investigated effects of a single-dose selective 5-HT reuptake inhibitor (escitalopram) on emotion regulation during virtual violence. For this purpose, 38 male participants played a violent video game during fMRI scanning. The SSRI reduced neural responses to violent actions in right-hemispheric inferior frontal gyrus and medial prefrontal cortex encompassing the anterior cingulate cortex (ACC), but not to non-violent actions. Within the ACC, the drug effect differentiated areas with high inhibitory 5-HT1A receptor density (subgenual s25) from those with a lower density (pregenual p32, p24). This finding links functional responses during virtual violent actions with 5-HT neurotransmission in emotion regulation networks, underpinning the ecological validity of the 5-HT model in aggressive behavior. Available 5-HT receptor density data suggest that this SSRI effect is only observable when inhibitory and excitatory 5-HT receptors are balanced. The observed early functional changes may impact patient groups receiving SSRI treatment.

**Electronic supplementary material:**

The online version of this article (10.1007/s00429-018-1693-2) contains supplementary material, which is available to authorized users.

## Introduction

### Cognitive emotion regulation

The neural circuitry of cognitive emotion regulation includes the orbitofrontal cortex (OFC), and lateral and medial prefrontal cortex (PFC), such as anterior cingulate cortex (ACC), and amygdala (Elliott et al. 2011; Kohn et al. 2014; Morawetz et al. 2017). The medial prefrontal and cingulate cortices exert an inhibitory top–down control over the amygdala (Ochsner and Gross 2005; Motzkin et al. 2015), thus reducing negative emotions and stress (Lederbogen et al. 2011). Both the lateral and medial PFC play a role in emotion regulation; the lateral PFC mediates the deliberate aspects of emotional regulation (Sarkheil et al. 2015) whilst the medial PFC subserves automatic processes (Phillips et al. 2003; Elliott et al. 2011). Under physiological conditions, the prefrontal–amygdala circuit mediates the inhibition of negative emotional responses when there is a need to suppress limbic reactivity. For instance, when emotional interference is decreased performance on cognitive tasks increases (Olivier et al. 1990; Schiller and Delgado 2010; Etkin et al. 2011). Deficient emotion regulation is a symptom of many psychiatric disorders and have been linked to disruptions in cortico-limbic circuits (Millan et al. 2012); among those are depression, anxiety (Mayberg 1997; Zilverstand et al. 2017), bipolar disorder (Townsend and Altshuler 2012), schizophrenia (Morris et al. 2012; van der Velde et al. 2015) and obsessive–compulsive disorder (OCD, Benzina et al. 2016). Although frontal cortex regions and amygdala are implicated in each of the aforementioned disorders and conditions, the dysfunctions vary. For instance, bipolar disorder is generally defined by a hyperactivity in the ventrolateral PFC during up- and downregulation of negative effect (Morris et al. 2012); this region is hypoactive in schizophrenia patients during reappraisal of emotions (van der Velde et al. 2015). Furthermore, connectivity between the dorsolateral PFC and the amygdala is reduced in patients suffering from major depressive disorder, schizophrenia and bipolar disorder (Millan et al. 2012; Morris et al. 2012; Zhang et al. 2018), indicating reduced ability for emotion control in these disorders. Interestingly, in OCD, which is characterized by intrusive thoughts and ritualized repetitive behaviors aimed at reducing thought-induced distress and anxiety (Rauch and Carlezon 2013; Maia and Cano-Colino 2015), the OFC, ACC and caudate display hyperactivation at rest and during symptom provocation (Benzina et al. 2016).

A dysfunctional fronto-amygdalar circuit has particularly been linked to emotion regulation deficits related to aggressive behavior, such as in overly aggressive individuals (Brower 2001; Bufkin and Luttrell 2005), to impulsive aggression (Davidson 2000), physical aggression connected to low self-esteem (Garofalo et al. 2016), negative affect (Donahue et al. 2014), and anger control (Velotti et al. 2017). While in intermitted explosive disorder, which is characterized by emotional outbursts and anger, activation in amygdala and medial PFC is enhanced (Coccaro et al. 2007), psychopathy is characterized by hypoactivation of these areas (Kiehl 2006; Decety et al. 2013).

Thus, fronto-limbic networks support the regulation of emotion and aggression required for cognitive and social functioning.

### Emotion regulation and serotonin (5-HT)

Dysfunctions in prefrontal–amygdala regulation have been attributed to the altered transmission of serotonin (5-HT; Bjork 2000; Buckholtz et al. 2008; Comai et al. 2012; Passamonti et al. 2012). 5-HT is a monoaminergic neurotransmitter synthesized by neurons originating in the raphe nuclei and projecting widely throughout the brain. The neurons particularly innervate the frontal and subcortical regions involved in emotion regulation; among those are the PFC (ventrolateral and ventromedial PFC, ACC), septum, basal ganglia, hippocampus, and amygdala (Fisher et al. 2009; Charnay and Léger 2010; Adell 2015). Accordingly, alterations in central nervous 5-HT signalling lead to dysfunctions in cognitive control and emotion regulation as seen in a range of neuropsychiatric disorders, including anxiety-spectrum disorders, OCD, depression, bipolar disorder, antisocial personality disorder and schizophrenia (Lesch 1998; Mahmood and Silverstone 2001; Bond 2005; Gaber et al. 2015). The most compelling evidence for a role of 5-HT in emotional and social behavior is that treatment with selective serotonin reuptake inhibitors [SSRIs; pharmacological domain according to NbN-2: serotonin; mode of action according to NbN-2: reuptake inhibitor (SERT)] is beneficial for patients with disorders of social and emotional control (Siever 2008; Elliott et al. 2011). Although long-term treatment effects vary greatly from single-dose effects (as applied in this study; see discussion for more detail), studies with treated patient groups gave important insights on the neurobiological mechanisms. It has been established that the neurotransmission of 5-HT plays a major role in emotion regulation during impulse control and aggressive behavior (Davidson 2000). Evidence for this aforementioned observation stems from rodent models and human PET/fMRI studies that directly measured or modulated the availability of proteins involved in serotonergic neurotransmission (e.g. Albert 2012; Buckholtz et al. 2008; Niederkofler et al. 2016). Furthermore, the impact of 5-HT has been directly tested with pharmacological interventions such as acute tryptophan depletion (Zimmermann et al. 2012; Kötting et al. 2013; Gaber et al. 2015; Eisner et al. 2017) and short-term or single-dose SSRI application (for a review see Anderson et al. 2008). Taken together, a wide range of behavioral and neurobiological studies corroborated the immense impact of 5-HT on social cognition, and particularly, on emotion regulation processes.

### Pharmaco-fMRI and SSRIs

By manipulating 5-HT function in the human brain, we can improve our understanding of the underlying neurobiological mechanisms of mood, anxiety and impulse control disorders. In pharmaco-fMRI, the serotonergic system can be manipulated pharmacologically with an SSRI, while brain fMRI data are collected simultaneously (Anderson et al. 2008; Klomp et al. 2013). One method of manipulating the serotonergic system (i.e., conducting a serotonergic challenge), is by administering a single dose of the SSRI escitalopram. Escitalopram functioning is well understood (for a review see Zhong et al. 2012). The mechanism by which escitalopram functions is that it blocks the 5-HT transporter and thus increases 5-HT availability in the synaptic cleft, which—among other effects—results in stimulation of inhibitory serotonergic auto- and heteroreceptors. Long-term treatment leads to adaptations in the 5-HT-receptor and -transporter expression and availability, desensitization effects, and synaptic outgrowth, ultimately leading to anti-depressive and anti-aggressive effects (Zhong et al. 2012; Harmer et al. 2017).

Recently, there have been efforts to better understand the immediate effects of SSRIs on cortical functioning in healthy individuals. There is evidence that administration of a single dose of an SSRI [most often (es-)citalopram] alters blood oxygenation level-dependent (BOLD) responses in PFC regions including ACC and ventrolateral PFC, and amygdala during tasks involving emotion regulation and emotion recognition (for review see Anderson et al. 2008). However, most studies have focused on explicit and implicit processing of emotional faces or pictures and yielded mixed results. Murphy and colleagues (2009) reported increased amygdala responses to implicit processing of fearful faces after the SSRI. This finding has been supported and extended in several similar studies; for example, citalopram (Bigos et al. 2008) and escitalopram (Cremers et al. 2016) increased amygdala reactivity during explicit facial emotion recognition. In contrast, amygdala activation was attenuated during implicit recognition of facial emotions (Del-Ben et al. 2005; Anderson et al. 2007). There is also evidence showing enhanced functional responses in prefrontal cortical areas following SSRI (insula: Anderson et al. 2007; OFC; Del-Ben et al. 2005; ACC & dorsolateral PFC; Rahm et al. 2014). However, research investigating brain activation following exposure to emotional pictures has yielded mixed results. Specifically, administration of citalopram was associated with an increased activation in the medial PFC, dorsolateral PFC and inferior frontal gyrus (IFG) during the presentation of positive, negative, and neutral pictures (Brühl et al. 2010). Similarly, intravenous citalopram increased activation in the left ACC during the presentation of neutral pictures compared to rest, and to happy compared to neutral pictures (Anderson et al. 2011). In a separate study, the administration of escitalopram also increased activation to positive pictures in the IFG; however, there were decreased responses to negative pictures (Outhred et al. 2014). During a Stroop task using emotional faces, citalopram decreased ACC and ventromedial PFC responses (Rahm et al. 2014). Taken together, SSRI treatment modulates prefrontal activation, but the exact effects are task dependent. In particular, dorsolateral PFC, medial PFC and ACC are strongly implicated.

In summary, pharmaco-fMRI studies using single-dose SSRIs have predominately focused on affect processing for emotional facial expressions and pictures, and results remain inconsistent. Furthermore, evidence from other affective challenges, particularly related to impulse and aggression control, remains under investigated.

To account for such divergent findings, regional and dynamic differences in SSRI effects must be considered. The ACC in particular seems to be sensitive to alterations in serotonergic neurotransmission in psychopathology. Pre- and subgenual ACC subregions respond differently to SSRIs; only the pregenual ACC, but not the subgenual ACC, has been linked to antidepressant treatment outcome (Mayberg 1997; Drevets et al. 2008). Regional differences in responses may be explained by varying 5-HT transporter and -receptor profiles. Depressed patients display a reduced 5-HT transporter availability in the ACC (Frankle et al. 2005) and patients with mutations in the serotonin transporter gene (5-HTTLPR) exhibit blunted responses to SSRI treatment (Pettitt 2015). Furthermore, receptor binding of the 5-HT_1B_ receptor is reduced in the ACC (Tiger et al. 2016). The 5-HT_1A_ receptor, in particular, shows altered functioning in clinical populations (Garcia–Garcia et al. 2014) and impacts SSRI functioning; SSRI efficacy varies with 5-HT_1A_ receptor activity (Altieri et al. 2013). Correspondingly, a low cortical 5-HT_1A_ receptor-binding potential is associated with non-remitters of severe depressive disorder (Parsey et al. 2006). With the help of cytoarchitectonical brain maps, variations of 5-HT receptor densities have been detected for the ACC subregions (Palomero-Gallagher et al. 2008, 2009). Indeed, 5-HT_1A_ receptor density is increased in the subgenual ACC compared to the pregenual ACC. Therefore, a region-of-interest (ROI) analysis may differentiate the contributions of ACC subregions on emotion regulation depending on their 5-HT receptor profile and function (Botvinick et al. 2004; Palomero-Gallagher et al. 2008, 2009).

### Virtual violence and emotion regulation

Directly studying the neural correlates of violent actions with functional imaging is difficult since experimental induction of aggressive acts is hardly feasible, both practically and ethically. However, virtual aggression in video games (e.g. killing virtual characters) provides a valid model for real-life aggression since they share behavioral responses (Bushman and Anderson 2002; Anderson et al. 2004; Adachi and Willoughby 2011) and neural substrates (Mathiak and Weber 2006; Cheetham 2009; Weber et al. 2009; Mathiak et al. 2011; Klasen et al. 2012b). It has been suggested that committing virtual violent actions during gameplay may elicit a guilt response (Hartmann et al. 2010; Grizzard et al. 2017) that depends on trait empathy and perceived immorality of the virtual action (Hartmann et al. 2010). In the study by Grizzard and colleagues (2017), the guilt response decreased after repeated exposure. This indicates the presence of emotion regulation processes during the experience of virtual violent actions and is in line with the observed negative correlations between clusters in the ACC and the amygdala with the violence construct in a first-person shooter game (Weber et al. 2009). The violence-dependent downregulation of ACC may serve as a mechanism to suppress task-irrelevant emotions causing empathy so that the individual could play the game successfully (Mathiak and Weber 2006; Weber et al. 2009). Furthermore, short scenes recorded from violent gameplay elicited less frontal brain activation in frequent players when compared to non-gamers, and this may relate to habitually reduced emotional processing (Regenbogen et al. 2010). In a study showing pictures from a standardized set (International Affective Pictures System (IAPS) catalog), negative valence elicited less activation in IFG in frequent players compared to non-gamers, possibly indicating reduced empathy in gamers (Montag et al. 2012). All of these studies used cross-sectional designs to provide evidence for an association between video-gaming and alterations in prefrontal activation in gamers. Thus, it remains uncertain whether a low prefrontal activation level predisposes for gaming behavior or whether habitual gaming reduces the general activation level. A more causal evidence for direct gaming effects on prefrontal responses is provided by a recent study by Zhou and colleagues (2017). By combining a cross-sectional with a longitudinal design, the study demonstrates gaming-induced decrease in the left orbitofrontal gray matter volume. A pharmaco-fMRI study directly tested the influence of short-term Quetiapine (atypical antipsychotic with serotonergic and dopaminergic properties) administration on neural processing during virtual aggression and reported increased functional connectivity of ACC and dorsolateral PFC with the amygdala (Klasen et al. 2013). This study provided first evidence of a possible serotonergic modulation of prefrontal–amygdala networks during virtual violence.

### Hypotheses

We tested whether 5-HT influences activation in the emotion regulation networks during virtual violence by challenging the serotonergic system using pharmaco-fMRI. It was hypothesized that if 5-HT was involved in emotion regulation during virtual violence, the administered SSRI would affect hemodynamic responses. In particular, we first replicated the finding that virtual violent action is associated with decreased functional responses in both the medial prefrontal brain regions and the amygdala. Second, we hypothesized that acute SSRI effects would reduce these violent-dependent hemodynamic responses in the emotion regulation networks, in particular, in medial PFC structures such as the ACC. Finally, we aimed to investigate the differential contributions of the pregenual and subgenual ACC regions, which differ in their 5-HT receptor profile and function (Botvinick et al. 2004; Palomero-Gallagher et al. 2008, 2009).

## Materials and methods

### Participants

The study was advertised with posters placed on notice boards at the University Hospital Aachen and other buildings of the RWTH Aachen University. Participants volunteering for the study were screened for inclusion and exclusion criteria before admission. Due to the differential effects of antidepressants on emotion processing for males and females (see e.g. Marazziti et al. 2014), only males were included in this study. In total, 38 male Caucasians (mean age 24.7 ± 3.6 years) participated in the experiment. All participants had normal or corrected to normal vision, normal hearing, no contraindications against magnetic resonance (MR) investigation, no history of neurological or psychiatric illness according to the Structured Clinical Interview for DSM-4 (SCID-4) screening questionnaire (Wittchen et al. 1997), and no history of psychopharmacological therapy. Further exclusion criteria were heavy smoking (more than eight cigarettes per day) and a positive drug screening test (urine dip test, MöLab GmbH, Langenfeld, Germany), which was administered immediately before the start of the study on each test day. Furthermore, acute medication, including hormonal treatment, was an exclusion criterion. All participants had regularly played video games before. 28 participants (74%) were habitual players at the time of the experiment with an average gameplay of 7.1 ± 9.5 [mean ± standard deviation (std)] hours per week. The most frequently played game categories were first-person shooters, strategy games and sports/ racing games. All participants were right handed according to the Edinburgh Handedness Inventory (Oldfield 1971). The average years of school education were 12.7 ± 0.09 years (mean ± std). Of the 38 participants, most were students completing a bachelor’s, a master’s or a doctoral program (31). The rest of them were undergoing vocational training (4), just finished school (2), or were currently unemployed (1). The experiment was designed according to the Code of Ethics of the World Medical Association (Declaration of Helsinki) and the study protocol was approved by the local Ethics Committee. Informed consent was obtained from all individual participants included in the study. Participants were financially compensated (10 €/h).

### Procedure

The study employed a randomized double-blind, placebo-controlled cross-over design. Each participant was measured on 2 days, i.e., once in the placebo (PLAC) condition and once in the drug (SSRI) condition. The measurements were separated by at least 1 week allowing for a washout of the drug. The condition order was randomized across the experimental group. In both conditions, the participants received a single dose of one pill 3 h prior to the fMRI measurements (placebo pill or the SSRI Cipralex^®^, 10 mg; Lundbeck GmbH, Hamburg, Germany). The drugs were block randomized (block size 6) and packed in coded blisters by the pharmacy who was not further involved in the experimental procedure. We chose a 3-h waiting period because in healthy, young adults taking a single 10 mg dose, the maximum plasma concentration is reached after 3.9 ± 1.8 h. The elimination half-time is 29.0 ± 14.2 h. (Rao 2007). By starting the fMRI measurement 3 h after drug intake we ensured that the complete 1.5 h measurement was within the phase of maximal plasma concentration. During the four scanning sessions (10 min each), the participants played the violent video game Carmageddon: TDR 2000 (Torus Games, Bayswater, Australia, 2000) in an unrestricted manner.

Carmageddon is a car racing game where the player drives a car through a virtual landscape. During the race, extra points can be gained by killing pedestrians or by collecting bonus items. The game sound environment was multi-layered with several sound effects occurring and intermingling. Besides the constant roaring motor sound of the driving car and the other cars, bonus point collection elicited a sparkling sound effect. Targeting or hitting a pedestrian elicited screams of the victims. A car crash of any kind led to a clashing and clattering sound.

Two modes of the video game were employed in the present study. In the standard version, the participants were instructed to kill as many pedestrians as possible. Hitting pedestrians would lead to an excessive depiction of blood splatter, accompanied by screams of the virtual victims. To allow for the occurrence of non-violent events, participants were also asked to play an altered version that contained no pedestrians and the participants were instructed to collect as many bonus items as possible. Each participant played two standard and two modified sessions of the game in a randomized order. This procedure allowed the experimental design to achieve approximately an equal number of violent and non-violent events.

Participants played the video game with an MR-compatible keyboard (Lumitouch). Visual stimulation and audio effects were delivered via MR-compatible video goggles and headphones; sound levels in the video game were individually adjusted to each participant’s comfort level. The video and the audio of the game play were recorded by a frame grabber (15 Hz frame rate, Fraps, Beepa) for subsequent content analysis.

### Data acquisition

Magnetic-resonance imaging was conducted on a 3 T MR Scanner (Magnetom Trio, Siemens) with a 12-channel head coil using echo-planar imaging (EPI) sequences [echo time (TE) = 28 ms, repetition time (TR) = 2000 ms, flip angle = 77°, voxel size = 3 × 3 mm, matrix size = 64 × 64, 34 transverse slices, 3 mm slice thickness, 0.75 mm gap]. For each measurement, a total of 1240 functional images were acquired (four sessions with 310 volumes) resulting in a total duration of 4 × 10 = 40 min. Anatomical images were recorded with a magnetization prepared rapid acquisition gradient echo (MPRAGE) pulse sequence yielding high-resolution T1-weighted anatomical images [TE = 2.52 ms, TR = 1900 ms, inversion time (TI) = 900 ms, flip angle = 9°, field-of-view (FOV) = 256 × 256 mm^2^, 1 mm isotropic voxels, 176 sagittal slices].

### Event definition and content analysis

**Fig. 1:**
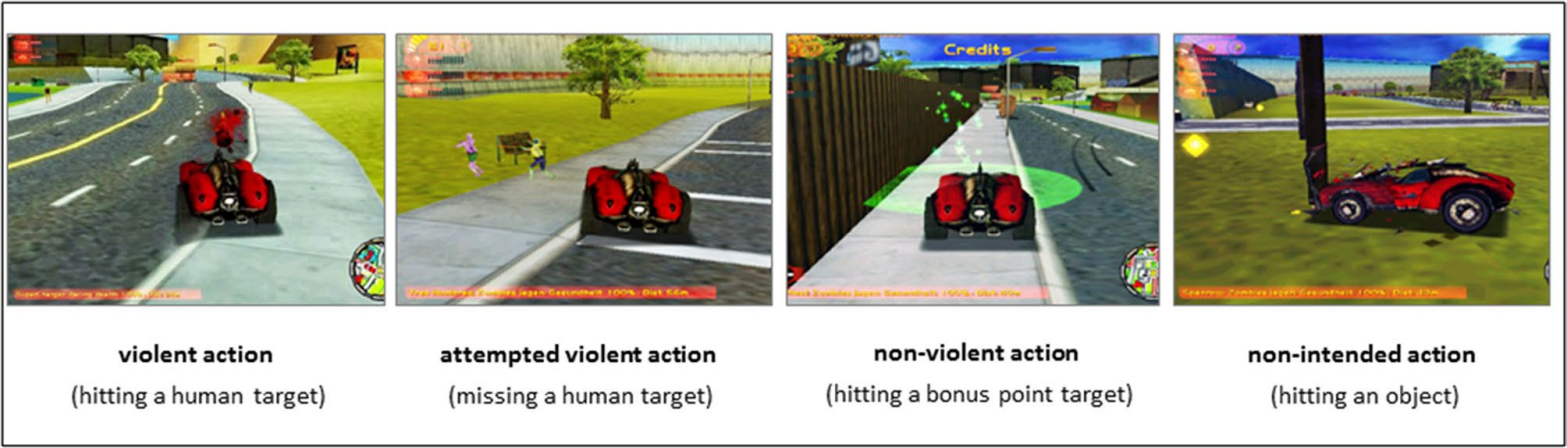
Gameplay event types. On each of the two measurement days, participants played the racing game *Carmageddon* during four 10-min fMRI sessions. During the gameplay, participants could run over and kill virtual pedestrians with their car (event type ‘violent action’; 1st panel). When participants narrowly missed the pedestrian ‘attempted violent action’ was coded (2nd panel). During the ‘non-violent action’ a bonus-item was collected (3rd panel). ‘Non-intended actions’ were coded when the player crashed into objects or scenery in the game map (4th panel). Video and sound of the gameplay were recorded; time point and type of each event were coded; and a response model was generated for fMRI analyses

Video game events were coded with the viewing and annotation software ELAN 3.8.0 (MPI for Psycholinguistics, Nijmegen, The Netherlands). Coding was conducted on a frame-by-frame basis with 67 ms accuracy according to the 15-Hz frame rate. Four event types were coded: violent action (killing a pedestrian), attempted violent action (attempted killing of pedestrian), non-violent action (collecting bonus items), and non-intended action (hitting an object; Fig. 1). The remaining gameplay, which mainly comprised of car-driving activity, served as baseline for the statistical analysis (see Sect. 2.5). This coding scheme yielded the time points of the identified actions and their type. Both violent actions and attempted violent actions were defined by the intention to kill virtually. Specifically, these actions differed in the actual event of hitting a pedestrian. The coders received intensive training and supervision on gameplay recordings not used in the study. The coding scheme gained inter-rater reliabilities of > 0.9 (Krippendorff’s Alpha) in pretesting. The video recordings entering the analysis were coded once by one coder. The event modelling yielded high time-resolution content analyses that were previously described and validated for fMRI applications (e.g. Weber et al. 2009; Mathiak et al. 2005). A two-factor analysis of variance (ANOVA, factor event with four levels and factor drug with two levels) assessed the event counts for a drug effect at the behavioral level (IBM SPSS, Statistics for Windows, Version 20.0, Armonk, NY: IBM Corp.).

### Image analysis

Statistical analysis of fMRI data was performed with BrainVoyager QX 2.4 (Brain Innovation, Maastricht, The Netherlands). Pre-processing included slice-scan time correction, spatial smoothing (4 mm full-width at half-maximum Gaussian kernel), 3D motion correction (alignment to the first volume of the first session, sinc interpolation), and high-pass filtering including linear trend removal. The first five images of each session were discarded to avoid T1 saturation effects. Subsequently, functional data were coregistered to the 3D anatomical image and transformed into Talairach space (Talairach and Tournoux 1988).

We constructed an event-related general linear model (GLM) from the coded events with separate predictors for each drug (PLAC, SSRI) and each event type (violent action, attempted violent action, non-violent action, non-intended action). The resulting model was convoluted with a standard hemodynamic response function. Statistical parametric maps were computed using the BrainVoyager ANCOVA module with the factors drug (two levels) and event (four levels). The analysis yielded three whole-brain maps: One for each factor and one for the interaction. Additionally, a t-contrast compared ‘violent actions’ to ‘non-violent actions’. All statistical maps were set with a threshold of voxel-wise *p* < 0.005 and cluster size *k* > 8 voxels, corresponding to a *p* < 0.05 corrected for multiple comparisons according to Monte Carlo simulations.

Effects of SSRI challenge on neuronal activations were further investigated by a functional ROI analysis. We defined functional ROIs from the clusters yielding significance in the whole-brain maps for the factor drug and for the interaction drug × event (see Fig. 3a). For each of the three clusters, a descriptive two-factor ANOVA differentiated the factors drug and event. To avoid double-dipping, only contrasts were considered that were orthogonal to those of the original maps. Post hoc t tests investigated drug effects for each event separately.

### Association with 5-HT receptor densities

The ACC is comprised of several subregions, which differ in cytoarchitecture and neurotransmitter-receptor densities (Palomero-Gallagher et al. 2008, 2009). Microanatomically-informed masks defined ROIs for the pregenual areas p24 and p32, and the subgenual area s25. Notably, these pre- and subgenual areas differ markedly in the density of the inhibitory 5-HT_1A_ receptor (Palomero-Gallagher et al. 2009, 2015). In these anatomical ROIs, average beta-values were extracted and entered a three-factor ANOVA (factors ROI with three, drug with two, and event with four levels). Since the factor ROI yielded significance, post hoc analysis further investigated the influence of drug and event with two-factor ANOVAs (drug with two and event with four levels). *t* tests separated drug effects between the events. Statistics for the ROI analyses (activation clusters and anatomical) were conducted using SPSS (IBM, Statistics for Windows, Version 20.0, Armonk, NY, USA: IBM Corp.).

## Results

### Behavioral effects

**Fig. 2:**
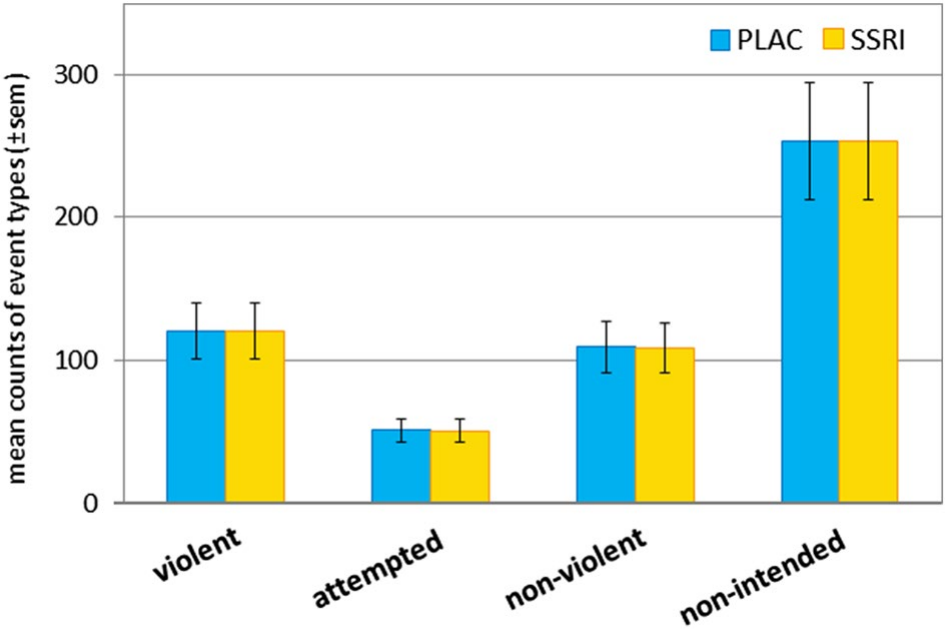
Mean count of coded event types. On each measurement day, participants played four 10-min sessions of the racing game Carmageddon. Gameplay was video recorded and subsequently coded with four event types (compare Fig. 1). A total of 40,548 events were coded throughout the study. The counts of violent actions and non-violent actions were similar. Similar event counts under PLAC and SSRI conditions suggest a high robustness of the behavioral paradigm: violent action under PLAC: mean count ± sem = 120.8 ± 32.5, SSRI = 120.5 ± 32.3; attempted violent action: PLAC = 50.8 ± 28.3, SSRI = 50.5 ± 27.8; non-violent action: PLAC = 109.1 ± 21.6, SSRI = 108.5 ± 23.2; non-intended action: PLAC = 253.6 ± 57.9, SSRI: 253.3 ± 55.8). *sem* standard error of the mean, *PLAC* placebo

A total of 40,548 events were coded throughout the study. Of those, 9,169 events were coded as violent action (241.3 ± 58.4 (mean ± standard deviation) events per participant), 3849 events as attempted violent action (101.3 ± 54.7 per participant), 8269 events as non-violent action (217.6 ± 40.0 per participant), and 19,261 events as non-intended action (506.0 ± 104.1 per participant). Thereby the counts of violent actions and non-violent actions were almost balanced (difference of the means: PLAC: *T*_37_ = 2.09, *p* = 0.043; SSRI: *T*_37_ = 2.05, *p* = 0.047). The mean count of each event across participants was very similar under PLAC and SSRI (see Fig. 2). Accordingly, the two-factor ANOVA analysis did not reveal a behavioral effect of the SSRI challenge on the event counts (drug: *F*_1,37_ = 0.021, *p* = 0.88, n.s.; drug × event interaction: *F*_3,111_ = 0.001, *p* = 1.00, n.s.).

### Neuroimaging

#### Effect of event on neuronal processing

**Fig. 3:**
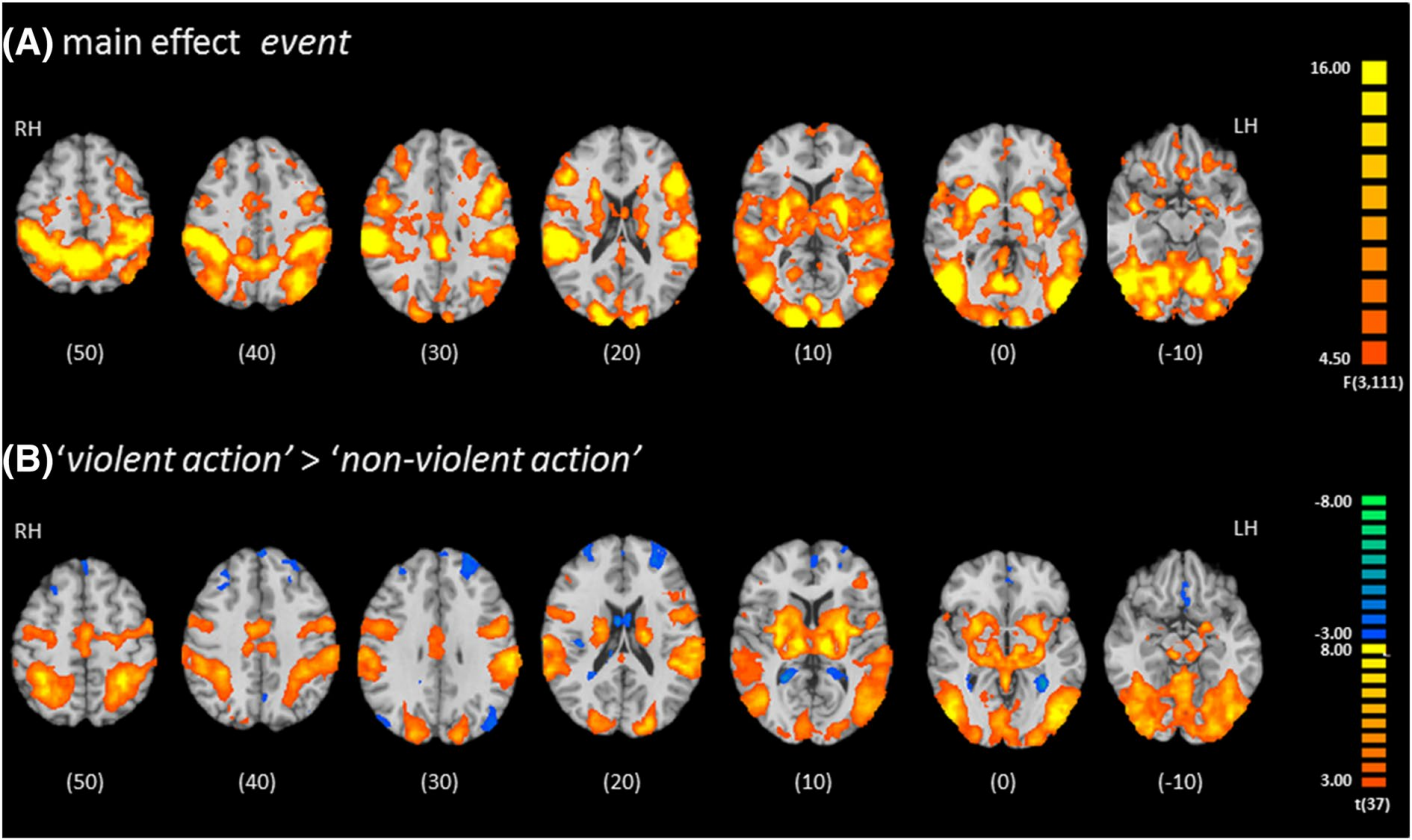
Effect of event type on functional responses. **a** The factor event (four game actions: violent, attempted violent, non-violent, and non-intended) explained variance in brain activity of extended sensory, motor, and reward networks (see Table 1). **b** Violent actions yielded similar distributed activation patterns and deactivation in the prefrontal cortex compared to non-violent actions. Maps were thresholded according to a corrected *p* < 0.05 after Monte Carlo simulation (voxel-wise *p* < 0.005 and cluster-size > 8). *Z* values are indicated beneath each slice

Considering the main effect event in the ANOVA analysis, the four gameplay events yielded strong activation changes in distributed neural networks covering large parts of the cerebral cortex as well as subcortical structures (putamen, striatum, and amygdala). The strongest responses were observed in the bilateral auditory and visual cortices, left frontal cortex, and the striatum (Fig. 3a; Table 1). The t-contrast between ‘violent action’ and ‘non-violent action’ revealed activation in similar subcortical structures as well as in the auditory, visual, and motor cortices. Furthermore, lateral and medial prefrontal cortices were deactivated during these events (Fig. 3b; Table 2).

**Table 1:** Brain regions affected by gameplay events (Fig. 3a)

Peak voxel location	Cluster size (voxel)	Peak *F* value	Peak voxel		
			x	y	z
Effects of event types					
LH putamen*	506,640	9.98	− 20	3	− 4
Extending into large parts of occipital, parietal, temporal and lateral frontal cortex					
RH insula	1116	6.99	34	21	2
Extending into inferior frontal gyrus					
LH anterior cingulate cortex^a^	3116	6.32	1	30	− 7
Extending into medial frontal gyrus					
LH medial frontal gyrus^a^	875	5.76	4	63	8
RH medial frontal gyrus^a^	762	5.46	4	30	47
LH superior frontal gyrus	570	5.60	− 11	39	47

Maps were thresholded at a voxel-wise *p* < 0.005 and cluster size *k* > 8 voxels, corresponding to a *p* < 0.05 corrected for multiple comparisons according to Monte Carlo simulations. Peak voxel coordinates are given in Talairach space

^a^Cluster extends to the other hemisphere

**Table 2:** ‘Violent action’ compared to ‘non-violent action’ (Fig. 3b)

Peak voxel location	Cluster size (voxel)	Peak *T* value	Peak voxel		
			*x*	*y*	*z*
Frontal cortex					
Left inferior frontal gyrus	11,992	− 6.67	− 50	30	14
Right insula	1524	− 5.86	40	− 12	2
Right anterior cingulate gyrus	1328	− 6.46	1	30	− 7
Left middle frontal gyrus	640	− 5.71	− 29	9	50
Left superior frontal gyrus	432	− 4.74	− 8	66	14
Left insula	351	− 4.53	− 35	− 21	− 4
Left precentral gyrus	336	− 4.12	− 56	− 3	11
Right medial frontal gyrus	313	− 4.59	1	54	2
Left middle frontal gyrus	303	− 4.35	− 44	3	41
Parietal cortex					
Right cuneus^a^	16,829	− 9.10	− 8	− 99	8
Extending into lingual, middle occipital, and fusiform gyrus					
Left angular gyrus	8566	− 6.11	− 35	− 60	38
Right gyrus supramarginalis	4631	− 6.63	58	− 24	32
Right posterior cingulate gyrus	2768	5.36	19	− 57	17
Left posterior cingulate gyrus	1881	− 6.27	− 5	− 39	32
Right precuneus	1333	5.12	16	− 66	41
Left gyrus supramarginalis	888	− 4.75	− 62	− 33	26
Left postcentral gyrus	788	4.93	− 38	− 24	47
Left precuneus	745	− 4.58	− 5	− 63	35
Right postcentral gyrus	639	6.19	22	− 33	50
Left precuneus	548	4.78	− 20	− 63	47
Temporal cortex					
Left fusiform Gyrus	5785	− 5.69	− 41	− 57	− 10
Extending into middle temporal gyrus					
Left lingual gyrus	417	− 4.52	− 2	− 69	2
Left fusiform gyrus	344	5.15	− 17	− 30	59
Occipital cortex					
Right middle occipital gyrus	761	5.32	55	− 69	3
Subcortex					
Right caudate*	5079	6.31	28	− 12	11
Extending into putamen					
Left caudate	2765	5.66	− 17	− 18	20
Extending into putamen					
Right thalamus	690	5.94	1	− 15	11
Left caudate	442	4.07	− 17	18	17

Maps were thresholded at a voxel-wise *p* < 0.005 and cluster size *k* > 8 voxels, corresponding to a *p* < 0.05 corrected for multiple comparisons according to Monte Carlo simulations. Cerebellar clusters are not reported. Peak voxel coordinates are given in Talairach space

^a^Cluster extends to the other hemisphere

#### SSRI effects and event types

**Fig. 4:**
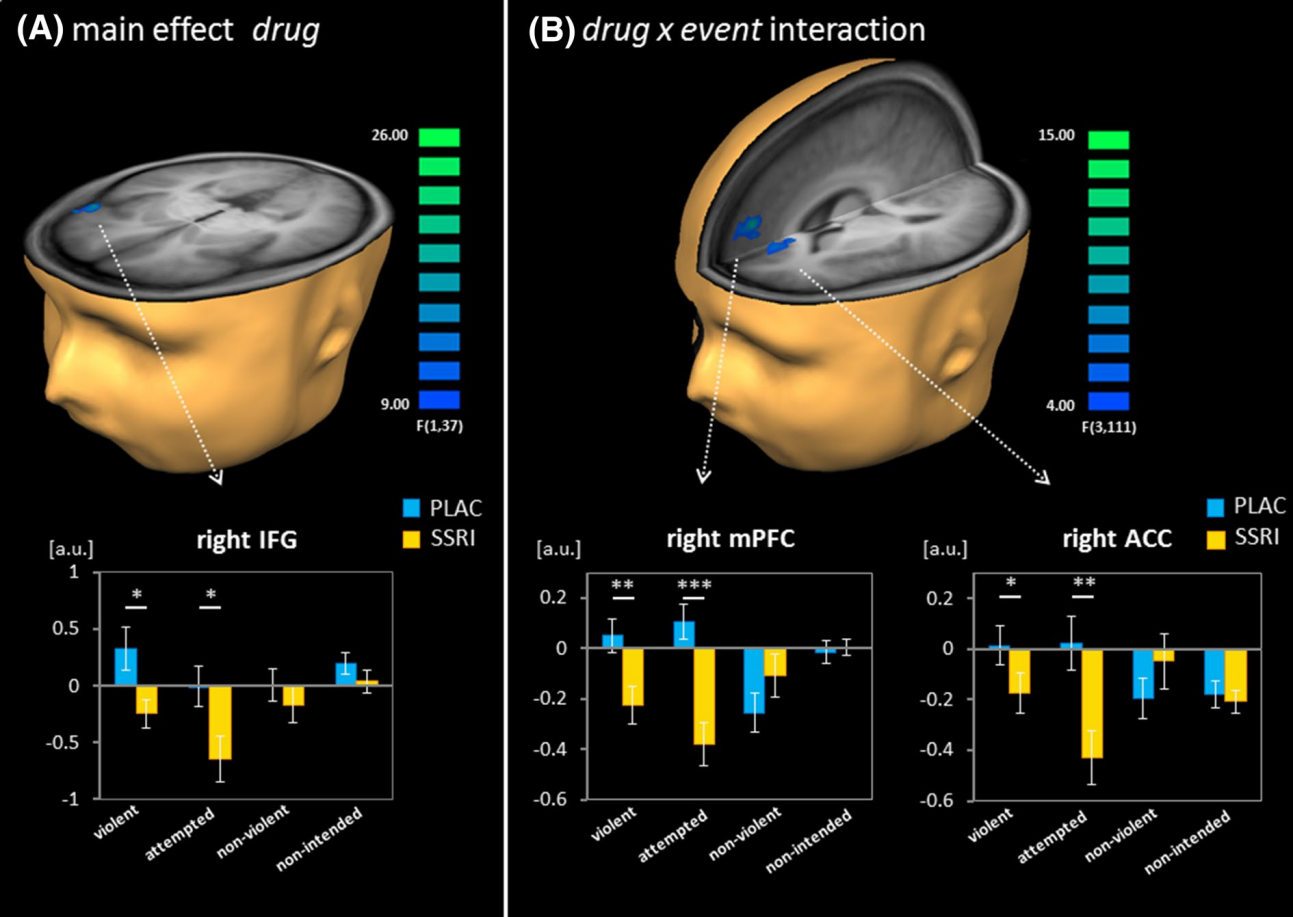
Effect of the SSRI challenge on functional responses. **a** The factor drug explained variance in the right inferior frontal gyrus (IFG). The SSRI challenge decreased responses to violent and to attempted violent actions but not to non-violent and to non-intended actions (see ROI analysis in bar graph). **b** The interaction between drug and event yielded significance in the right medial prefrontal gyrus (mPFC) and the right anterior cingulate cortex (ACC). In these two clusters, the SSRI challenge reduced functional activation to violent actions and attempted violent actions only (see bar graph inserts). Maps were thresholded according to a corrected *p* < 0.05 after Monte Carlo simulation (voxel-wise *p* < 0.005 and cluster size > 8). Post hoc *t* tests: **p < 0.01; *p < 0.05. *ACC* anterior cingulate cortex, *IFG* inferior frontal gyrus, *mPFC* medial prefrontal cortex, *PLAC* placebo

The whole-brain map for the factor drug revealed that the acute SSRI challenge altered functional responses in the right IFG (ANOVA main effect drug, Fig. 4a; Table 3). In the ROI analysis of this cluster, the factor event yielded significance (*F*_3,111_ = 3.46, *p* = 0.032) but not the drug × event interaction (*F*_3,111_ = 1.45, *p* = 0.232, n.s.). Post hoc *t* tests revealed that the SSRI challenge decreased responses in the IFG for the two violent events ‘violent action’ (*t*_37_ = 2.65, *p* = 0.012) and ‘attempted violent action’ (*t*_37_ = 2.44, *p* = 0.019) but not on a significant level for the non-violent events ‘non-violent action’ and ‘non-intended action’ (both p > 0.1).

**Table 3:** Effects of single-dose SSRI

Peak voxel location	Cluster size (voxel)	Peak *F* value	Peak voxel		
			*x*	*y*	*z*
SSRI effects (Fig. 4a)					
Right inferior frontal gyrus	419	10.96	52	21	− 1
Interaction drug × event (Fig. 4b)					
Right medial frontal gyrus	1284	6.05	4	48	26
Right anterior cingulate cortex	372	5.61	1	39	8

Maps were thresholded at a voxel-wise *p* < 0.005 and cluster size *k* > 8 voxels, corresponding to a *p* < 0.05 corrected for multiple comparisons according to Monte Carlo simulations. Peak voxel coordinates are given in Talairach space

^a^Cluster extends to the other hemisphere

The whole-brain mapping of the drug x event interaction localized event-specific SSRI effects. Two significant clusters emerged in the right medial PFC area and were labelled as mPFC and ACC according to the anatomy toolbox (Eickhoff et al. 2005; see Fig. 4b; Table 3). In the ROI analysis of the averaged beta-values in these clusters, significant drug effects as well as drug–event interactions emerged (mPFC: drug: *F*_1,37_ = 6.09, *p* = 0.018; drug × event interaction: *F*_3,111_ = 13.51, *p* < 0.001; event: *F*_3,111_ = 2.19, *p* = 0.093 (trend level); ACC: drug: *F*_1,37_ = 4.55, *p* = 0.040; drug × event interaction: *F*_3,111_ = 6.84, *p* = 0.001; event: *F*_3,111_ = 0.90, *p* = 0.431, n.s.). Additionally, post hoc *t* tests comparing the SSRI and placebo conditions for each event type revealed significantly decreased responses for violent actions (mPFC: *t*_37_ = 3.03, *p* = 0.004; ACC: *t*_37_ = 2.13, *p* = 0.040) and attempted violent actions (mPFC: *t*_37_ = 4.86, *p* < 0.001; ACC: *t*_37_ = 3.28, *p* = 0.002), but not for the non-violent events (all *p* > 0.1). Thus, in all the considered clusters, the single dose of SSRI reduced responses to violent events only.

Both whole-brain contrasts (‘main effect violence’ and ‘violent action > non-violent action’) revealed a strong activation in the putamen (Fig. 3), a region strongly implicated in reward processing. The processing of reward and anticipation is generally thought to be mediated by dopamine, particularly in the basal ganglia system (Nakamura 2006; Schultz 2016). However, accumulating evidence suggests a role of serotonin, as well (Kranz et al. 2010; Fischer and Ullsperger 2017). Since virtual violence in video games is a rewarding experience (Koepp et al. 1998; Klasen et al. 2012a; Zvyagintsev et al. 2016), the putamen may be effected by the SSRI during virtual violence, as well. Therefore, as an additional analysis, we calculated two whole-brain contrast which shed light on putamen responses. The first contrast compared ‘violent action’ with ‘attempted violent action’ to reveal reward anticipation-specific activation (Fig S1A). The second contrast demonstrated violent-specific activation by calculating a conjunction for violent action against the other three events (for a detailed description of methods see supplement S1). The contrast ‘violent action’ > ‘attempted violent action’ revealed distinct activation clusters in the putamen, among others (Fig. S1A, Tab. S1). Notably, activation was increased in lateral PFC areas, but not in the medial PFC.

The conjunction contrast for violent action revealed distinct activation clusters in the left and right putamen (Fig. S1B, Tab. S1). In both clusters, the subsequent ROI analysis demonstrated reduced activation after the SSRI only for violent action but not for the other events; notably also not for attempted violent action (Fig. S1B; for detailed results description see supplement S1).

#### SSRI challenge and ACC subregions

**Fig. 5:**
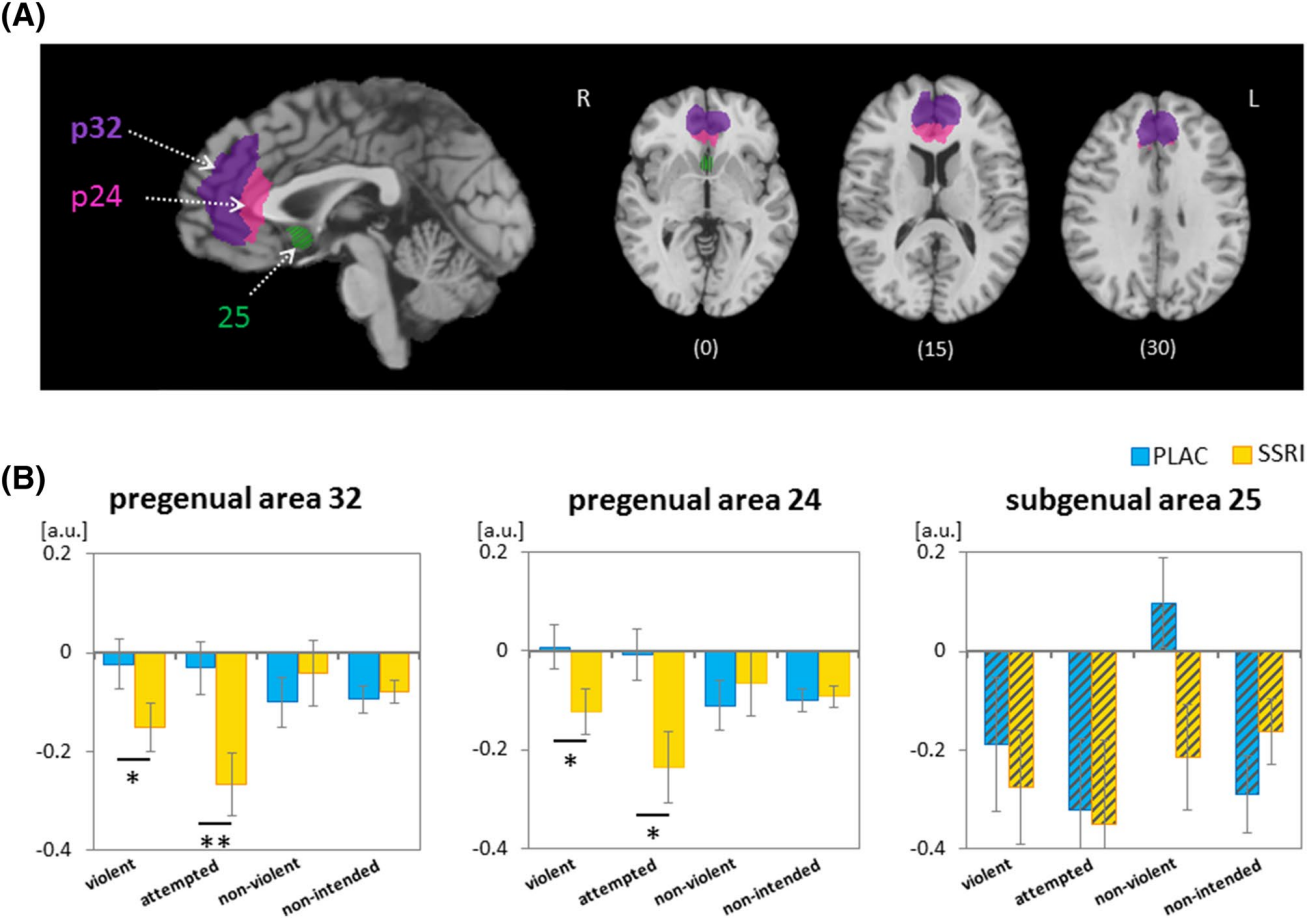
Effect of the SSRI challenge on functional responses in ACC subregions. The pregenual ACC subregions p32 and p24, and the subgenual region s25 were defined anatomically based on their cytoarchitecture (Palomero-Gallagher et al. 2008); p24 and p32 exhibited average and s25 elevated 5-HT_1A_ receptor density (Palomero-Gallagher et al. 2009). *Z* values are indicated beneath each slice. (B) The SSRI challenge enhanced deactivation in the pregenual subregions p32 (left panel) and p24 (middle panel) to violent action and to attempted violent action but not to non-violent or to non-intended actions. In the subgenual s25, the SSRI yielded no significant effect (right panel). Post hoc *t* tests: **p < 0.01; *p < 0.05. *PLAC* placebo

The subsequent microanatomically-informed anatomical ROI analysis of the ACC differentiated the pregenual p24 and p32 as well as subgenual subregion s25 (Fig. 5a). Average beta-values were extracted from each anatomical ROI and modelled with the three factors ROI, drug, and event in an ANOVA. The main effect ROI yielded significance (*F*_2,74_ = 5.08, *p* = 0.009), confirming subregion specific effects. The main factor drug and the triple interaction ROI × drug × event yielded a trend effect (drug: *F*_1,37_ = 3.09, *p* = 0.087; ROI × drug × event: *F*_6,222_ = 2.67; *p* = 0.062; main effect event and all other interactions *p* > .01).

In the post hoc assessment, ANOVAs tested the factors drug and event in each of the ROIs. In both pregenual regions p24 and p32, the main effect drug (p24: *F*_1,37_ = 4.17, *p* = 0.048; p32: *F*_1,37_ = 3.08, *p* = 0.088, trend) and the interaction drug × event yielded significance (p24: *F*_3,111_ = 3.61, *p* = 0.029; p32: *F*_3,111_ = 5.02, *p* = 0.006). In the subgenual region s25, no such effect or interaction was observed (all *p* > 0.1). The factor event failed to reach significance in all subregions (all *p* > 0.1). The post hoc *t* tests discerning drug effects in areas p24 and p32 revealed a significant reduction of regional responses under SSRI challenge for the two violent events ‘violent action’ (p24: *t*_37_ = 2.22, *p* = 0.033; p32: *t*_37_ = 2.10, *p* = 0.043) and ‘attempted violent action’ (p24: *t*_37_ = 2.64, *p* = 0.012; p32: *t*_37_ = 3.06, *p* = 0.004; Fig. 5b) only. Thus, the SSRI effects differed across ROIs, where the pre- but not the subgenual ACC subregion responded less after the SSRI challenge compared to placebo; in the area with high 5-HT_1A_ receptor density (s25), the SSRI effect was absent.

## Discussion

We investigated acute SSRI effects on functional responses to virtual violent and control (non-violent) actions. The SSRI reduced responses to violent actions in right-hemispheric IFG and two mPFC clusters, one of which is located in the pregenual ACC. In the latter clusters, this effect was specific to the violent gameplay events ‘violent action’ and ‘attempted violent action’. Thus, core structures for emotion regulation were modulated by post-synaptic 5-HT availability. At the ACC, the drug effect differentiated areas with a high density of inhibitory 5-HT_1A_ receptor (subgenual s25) from those with a lower density (pregenual p32 and p24). This pharmaco-fMRI study provides evidence of a link between functional responses to virtual violent actions and serotonergic neurotransmission in emotion regulation networks (mPFC including the ACC) and contributes a model to investigate emotion regulation during violent actions.

### Neural correlates of virtual violence

During the execution of virtual violent actions, the hemodynamic activity decreased in the lateral and medial PFC, including the ACC and the OFC. In contrast, the activity increased in the midcingulate cortex and widespread networks covering temporal, parietal, and occipital cortical areas, as well as the basal ganglia as part of the reward system. This pattern of functional responses during violent gameplay replicates findings from a study using the same game (Klasen et al. 2013) and mirrors findings from first-person shooter games (Mathiak and Weber 2006; Mathiak et al. 2011, 2013). Thus, neural processing during the violent car racing game may share features with first-person shooter games, and emotion regulation in both scenarios may be comparable. ACC activity was suppressed during the experience of virtual violence, which is thought to reflect reduced limbic responses and emotional interference during the gameplay (Mathiak and Weber 2006). In contrast to the study on the first-person shooter, amygdala suppression to violent actions failed significance during the racing game. Indeed, it was previously hypothesized that in fear-free environments, violent actions may not lead to BOLD changes of the amygdala (Weber et al. 2009). In contrast to first-person shooters, the avatars in the racing game did not attack or kill the player’s character. Even if the car was wrecked during a crash, it was immediately reset and gameplay continued. This lack of threat may have prevented high-valence responses such as fear and anxiety. Finally, the game was played in a non-competitive way as the participants were free to roam the map while ignoring the race. In a study on four video games which varied in violent content and competitiveness, only competitiveness increased arousal as measured by heart rate (Adachi and Willoughby 2011). Virtual killing may induce complex emotions such as guilt and empathy, which implicate contributions from mPFC. In contrast, basic emotions—such as fear—recruit structures such as the amygdala and insula (Elliott et al. 2011; Bernhardt and Singer 2012). In summary, the observed activation patterns to gameplay events of Carmageddon are in line with previous research and confirm a valid analysis strategy.

### Effects of SSRI challenge on brain activity

#### Effects of SSRI challenge on PFC activity and implication for emotion regulation

A single dose of the SSRI reduced hemodynamic responses to violent and attempted violent actions in the right IFG and two mPFC clusters. The finding supports our hypothesis that the central nervous 5-HT signalling is involved in prefrontal emotion regulation during virtual violence (Montag et al. 2012; Regenbogen et al. 2010). A reduction of emotional responses during gameplay is likely to be associated with diminished feelings, such as guilt or empathy (Hartmann et al. 2010; Grizzard et al. 2017). In turn, these cognitions have the potential to interfere with the task of successful gameplay (Mathiak and Weber 2006). The finding that the SSRI reduced activation suggests a further dampening of emotional responses as a result of the altered 5-HT signalling. Remarkably, the SSRI effected lateral and medial PFC responses similarly for both violent action and attempted violent action, but not for non-violent events or crashes. Likewise, significant deactivation in the medial PFC occurred for both violence-related actions. Attempted violence depicted an intention to kill an avatar, but the goal was not achieved. Thus, emotion processing may have been initialized prior to the actual event (cf. von Scheve 2012).

Repeated playing of violent video games can cause emotional desensitization (Anderson et al. 2004; Carnagey and Anderson 2005; Grizzard et al. 2017), which may be the result of increased emotion regulation. By further dampening emotional responses, the SSRI may aggravate emotional blunting during virtual violence. Although a single dose of an SSRI generally does not alter behavioral responses, a long-term treatment has been implicated in emotional blunting in patients receiving SSRIs (Barnhart et al. 2004; Goodwin et al. 2017). Therefore, long-term treatment may exacerbate gaming-related emotional blunting and may induce behavioral changes as well.

SSRI modulation of emotion regulation may not be confined to the experience of virtual violence. In a similar vein, single-dose SSRI lowered BOLD responses to affective interference in a Stroop task in the mPFC including the ACC (Rahm et al. 2014). The deactivations in the medial frontal cortex were associated with reduced emotional interference during the focused attention and problem-solving tasks associated with the Stroop. Although Rahm and colleagues reported an involvement of the bilateral IFG during the affective Stroop task, the SSRI did not alter activity in this lateral PFC area. Comparable to our study, the intake of a single-dose SSRI reduced the hemodynamic response in the right IFG during the observation of neutral and positive pictures (Outhred et al. 2014). The IFG is involved in several active strategies for emotion regulation (Kohn et al. 2014); thus, the decreased IFG activation may reflect modulation of reappraisal, which facilitates a positive information processing bias (Outhred et al. 2014). Outhred and colleagues (2014) further reported that the SSRI increased IFG responses to pictures with negative valence. The latter result seems to conflict with our finding of reduced activation during virtual violence. However, the playing of video games, including those with violent content, may be considered as a rewarding experience and, therefore, the events may not be appraised with negative valence (Koepp et al. 1998; Klasen et al. 2012b; Zvyagintsev et al. 2016). In summary, our data are in line with impaired emotion regulation after violent actions, and emotion appraisal and active reappraisal after a 5-HT challenge.

#### Effects of SSRI challenge on putamen and implications for reward processing

Playing video games is a rewarding experience (Koepp et al. 1998; Klasen et al. 2012b; Zvyagintsev et al. 2016); this is reflected in the increased activation in the reward system (putamen, insula, orbitofrontal cortex) as shown in the whole brain contrasts. The impact of violent action on the reward system was demonstrated by the strong putamen activation for violent actions compared to the other action types. Therefore, in addition to emotion regulation, reward processing may contribute to the neural responses.

The construct ‘reward anticipation’ can be indirectly investigated with our study design. Both violent action and attempted violent action were defined by the intention to kill virtually and only differed in the result (success vs. failure). The direct comparison of these two events should yield neural correlates for confirmed vs. disappointed expectation after anticipation of a virtual kill. Accordingly, the comparison ‘violent action > attempted violent action’ revealed potential effects of reward anticipation on our findings. In particular, a region in the right PFC overlapping with the reported cluster (main effect drug) was activated stronger for violent actions compared to attempted violent actions. Thus, reward anticipation may have interfered with emotion regulation. However, no such activation was detectable in the medial PFC areas.

The SSRI reduced activation in both the left and right putamen only for violent actions. Importantly, attempted violent actions, which also elicit reward anticipation, did not differ between the SSRI and placebo condition.

Although dopamine is the neurotransmitter commonly implicated in reward processing, serotonergic neurotransmission strongly influences reward-related processing such as reward value and motivational aspects (e.g. Kranz et al. 2010; Fischer and Ullsperger 2017). Therefore, the SSRI may have impacted both serotonergic and dopaminergic neurotransmission during virtual violent actions. This is particularly the case in the right lateral PFC, where reward anticipation seems to impact activity levels, and in the putamen, which differentiated confirmed and disappointed anticipation. In contrast, the SSRI affected both violent action and attempted violent action in the medial PFC and ACC, which hints at a more prominent serotonergic neurotransmission.

### Effect of short-term serotonergic challenge on the serotonergic system

In the current study, the serotonergic challenge by an SSRI reduced hemodynamic responses in the prefrontal cortex during virtual violent actions. Since the hemodynamic response mainly reflects local synaptic activity within a brain region (Logothetis 2002), we considered that violence-related processing in the PFC was mediated by serotonergic neurons or by neurons affected by serotonergic neurotransmission. The differential SSRI effects at the pre- and subgenual ACC provide further evidence of such serotonergic involvement. In contrast to the pregenual regions (p24, p32), the subgenual ACC (s25) exhibited lower hemodynamic responses during virtual violent actions in the placebo condition. Furthermore, SSRI application did not alter the subgenual responses. This difference in response profiles is reflected by the receptor densities; specifically in the subgenual region, the density of 5-HT_1A_ receptors is clearly above the cingulate cortex average (Palomero-Gallagher et al. 2009). The 5-HT_1A_ receptor expressed in the ACC is a post-synaptic, inhibitory heteroreceptor (Garcia–Garcia et al. 2014; Albert et al. 2014) and thus can be expected to increase post-synaptic inhibition of the serotonergic signal. Therefore, the local responses would be lower during virtual violent actions. High 5-HT_1A_ receptor density may protect the system against short-term dysregulation of 5-HT availability (Popova and Naumenko 2013).

The consequences of this differential response to the SSRI may be reflected in patients with mood disorders as well: only the pregenual ACC activity is linked to treatment outcome, while the subgenual ACC activity correlates positively with the severity of depressive symptoms (Mayberg et al. 1997; Drevets et al. 2008). Furthermore, an interaction of the 5-HT_1A_ receptor with SSRI efficacy is well established (Altieri et al. 2013). In particular, a high receptor-binding potential of 5-HT_1A_ has been linked to blunted treatment response in depressive patients (Parsey et al. 2006). In a similar vein, 5-HT_1A_ is implicated in the ethology and treatment of mood and anxiety disorders as well as in symptoms involving impulsivity and aggression in animal models and humans (Albert 2012; Altieri et al. 2013; Garcia–Garcia et al. 2014; Alekseyenko and Kravitz 2014). In summary, this study contributes to the increasing evidence that a single dose of SSRI alters prefrontal emotion regulation networks, which may reduce functional and affective responses to virtual violence.

### Single-dose vs. long-term SSRI treatment

Although models for long-term SSRI actions, i.e., increased levels of 5-HT and neuroadaptive changes, are well established (for a review, see Zhong et al. 2012), the effect of a single-dose SSRI on neural activity remains controversial. SSRI reuptake into the brain can be measured within a couple of hours; however, the treatment effectiveness is delayed for 2–4 weeks (Blier 2001). Following their rapid absorption SSRIs block the terminal 5-HT transporter. This impedes 5-HT reuptake into the pre-synapse and thus increases 5-HT levels in the synaptic cleft (Sharp et al. 1997). This imbalance is, however, countered by protective measures of the serotonergic system. With increasing 5-HT levels, the terminal 5-HT autoreceptors increasingly block 5-HT release into the synaptic cleft. This blocking may take place in the projection areas or at the level of cell bodies in the midbrain; the latter leading to a system-wide shut down (Nord et al. 2013; Garcia–Garcia et al. 2014). Therefore, although reuptake from the synaptic cleft is blocked, terminal 5-HT availability is decreased after SSRI intake (Blier 2001). With long-term administration of SSRIs, the autoreceptor gets desensitized (Piñeyro and Blier 1999). Furthermore, neuroadaptive changes modulate 5-HT neurotransmission to adapt to the new homeostasis with SSRIs present. For instance, desensitization effects and synaptic outgrowth as well as adaptations in serotonin receptor, and transporter expression and availability emerge (Piñeyro and Blier 1999; Zhong et al. 2012; Harmer et al. 2017). Therefore, therapeutic effect emerges because (a) the autoreceptors slowly desensitize, which allows the 5-HT neurons to progressively recover their normal firing rate, and, (b) the decreased density of the 5-HT transporters remains even after drug washout and continuously decreases 5HT clearance from extracellular space (Blier and Tremblay 2006; Garcia–Garcia et al. 2014).

Due to the complex neuroadaptive changes, the effect of SSRIs is highly dependent on treatment duration. In particular, a single dose may have an opposite effect on serotonergic signalling than a short- or long-term treatment (Murphy et al. 2009; Simmons et al. 2009; Di Simplicio et al. 2014). After a single-dose SSRI, only the fast neuroadaptive changes take place, that is, the system-wide shut down due to autoinhibitory processes at the cell bodies in the raphe nuclei. For instance, three hours after escitalopram treatment, brain 5-HT levels have been found to be reduced in the cortical projection areas (Nord et al. 2013). In this light, the lower BOLD response after the SSRI during virtual violent action may be explained by a generally reduced availability of 5-HT.

### Implication for mood disorders and their treatment

A single-dose SSRI may bias cognition, emotion regulation, and emotion recognition in healthy participants (Harmer et al. 2003; Alves-Neto et al. 2010) and depressed patients (Bhagwagar et al. 2004; Campbell 2008). Even in cases where no direct behavioral effects were detectable, a single-dose SSRI altered neural recruitment patterns (Rahm et al. 2014). So far, only few studies have investigated neural changes after single-dose SSRI application in patient populations; reasons may be that long-term effects were considered clinically more relevant. Short-term treatment (7 days) provides an intermediate procedure for the investigation of serotonergic contributions in emotion processing. After a 7-day SSRI treatment, later treatment responders had greater reduction in neural activity to fearful faces in a network including the ACC and the amygdala compared to non-responders (Godlewska et al. 2016). The ACC is the only region that may be used to differentiate eventual responders and non-responders for various depression treatments, including escitalopram (Pizzagalli 2011). Since we found the protective effect mediated by 5-HT_1A_ heteroreceptors to be particularly evident in the subgenual ACC, drugs targeting the 5-HT_1A_ receptor may increase efficacy in the case of patients not responding to SSRI treatment (Albert 2012; Albert et al. 2014; Pettitt 2015). Drugs with agonist activity at the 5-HT_1A_ receptor may also be of interest in the treatment of other mood disorders such as impulsivity and aggression (Takahashi et al. 2011; Bortolato et al. 2013). However, these considerations exceed the scope of the present study and, therefore, remain speculative.

Our study found that a single dose of SSRI altered brain functioning in healthy participants and similar effects may emerge in patients with mood disorders as well. In particular, short-term SSRI treatment increases emotion regulation but potentially reduces sensitivity to violent actions. Importantly, early modulations are essential for and may predict long-term treatment success. Further studies on these early effects should consider 5-HT receptor changes in addition to the 5-HT transporter inhibition.

## Limitations

The choice of a semi-naturalistic virtual paradigm opens novel possibilities of combining complex, dynamic social cues resembling real-life situations (Zaki and Ochsner 2009) but limits the control over stimuli. In particular, the number of experienced events remains unbalanced. In an attempt to equal the numbers, we introduced the control condition without humans on the map. This led to an alignment of event numbers for violent and non-violent action (violent action: 120.6 ± 13.8 events; non-violent action: 108.8 ± 12.5 events). Nevertheless, the unequal number of events may have contributed to differences in habituation and neural responses. Furthermore, the events were not balanced with respect to visuo-motor challenge and experience of arousal or reward/frustration. Violent and non-violent actions differed in the requirement of visuo-motor integration since the avatars moved, while the bonus points were stationary. Despite of these differences between events, the paradigm was very reliable and reproduced previous findings; the average event counts revealed almost identical event numbers under the placebo and the SSRI conditions. Thus, the drug did not affect game performance as a whole and the game environment afforded a stable stimulus.

An analysis of head motion differences between standard sessions (including violent events) and modified sessions (no violent events) revealed a larger amount of head motion for standard sessions for all parameters (translation and rotation on *x, y* and *z* axis). This difference was significant but very small (all translation and rotation differences equal to or smaller than 0.01 mm and 0.01 degrees, respectively).

On a neurobiological level, escitalopram is considered more selective and efficacious than other antidepressants (Zhong et al. 2012) but may affect dopaminergic and glutaminergic neurons as well; for instance by increasing their firing rate and burst firing (Alves-Neto et al. 2010). The BOLD response reflects overall energy consumption and enables only indirect conclusions on specific molecular mechanisms of 5-HT neurotransmission, in particular, in relation to 5-HT_1A_ densities.

Due to the sex difference of antidepressant effects on emotion processing, we only included male participants in this study. Therefore, our results may not be applicable to females.

## Conclusions

The regulation of emotions in the context of violence is modulated by 5-HT. This study shows, for the first time, that short-term inhibition of 5-HT reuptake reduces activity in the PFC nodes of emotion regulation during virtual violent action. Available 5-HT receptor density data suggest that this SSRI effect is only observable when inhibitory and excitatory 5-HT receptors are balanced. This study underpins the ecological validity of the 5-HT model in aggressive behavior. The single-dose SSRI effect indicated early functional changes in the emotion regulation network which may trigger SSRI treatment effects.

## Electronic supplementary material

Below is the link to the electronic supplementary material.

Supplementary material 1 (TIF 265 KB)

Supplementary material 2 (DOCX 23 KB)
