# Correction to: Central serotonin modulates neural responses to virtual violent actions in emotion regulation networks

**DOI:** 10.1007/s00429-019-01927-4

**Published:** 2019-08-07

**Authors:** Dhana Wolf, Martin Klasen, Patrick Eisner, Florian D. Zepf, Mikhail Zvyagintsev, Nicola Palomero‑Gallagher, René Weber, Albrecht Eisert, Klaus Mathiak

**Affiliations:** 1 Department of Psychiatry, Psychotherapy, and Psychosomatics, Medical Faculty, RWTH Aachen, Pauwelsstraße 30, 52074 Aachen, Germany; 2 Centre and Discipline of Child and Adolescent Psychiatry, Psychosomatics and Psychotherapy, Division of Psychiatry and Clinical Neurosciences and Division of Paediatrics and Child Health, School of Medicine, The University of Western Australia, Perth, Australia; 3 Specialised Child and Adolescent Mental Health Services, Department of Health in Western Australia, Perth, Australia; 4 Institute of Neuroscience and Medicine, Research Centre Jülich (INM‑1), Jülich, Germany; 5 Media Neuroscience Lab, Department of Communication, University of California Santa Barbara, Santa Barbara, CA USA; 6 Department of Pharmacy, RWTH Aachen, Aachen, Germany; 7 Department of Pharmacology and Toxicology, RWTH Aachen, Aachen, Germany; 8 JARA-Translational Brain Medicine, Aachen, Germany

## Correction to: Brain Structure and Function (2018) 223:3327–3345 10.1007/s00429-018-1693-2

The article “Central serotonin modulates neural responses to virtual violent actions in emotion regulation networks”, written by Dhana Wolf, Martin Klasen, Patrick Eisner, Florian D. Zepf, Mikhail Zvyagintsev, Nicola Palomero‑Gallagher, René Weber, Albrecht Eisert, Klaus Mathiak was originally published electronically on the publisher’s internet portal (currently SpringerLink) on June, 08, 2018 without open access.

With the author(s)’ decision to opt for Open Choice the copyright of the article changed July 2019 to © The Author(s) 2019 and the article is forthwith distributed under the terms of the Creative Commons Attribution 4.0 International License (http://creativecommons.org/licenses/by/4.0/), which permits use, duplication, adaptation, distribution and reproduction in any medium or format, as long as you give appropriate credit to the original author(s) and the source, provide a link to the Creative Commons license and indicate if changes were made.

