# Supplementary figures and images for: Central serotonin modulates neural responses to virtual violent actions in emotion regulation networks
Source: Brain Struct Funct. 2018 Jun 8;223(7):3327–45. doi: 10.1007/s00429-018-1693-2 (PMC6698268; doi:10.1007/s00429-018-1693-2)

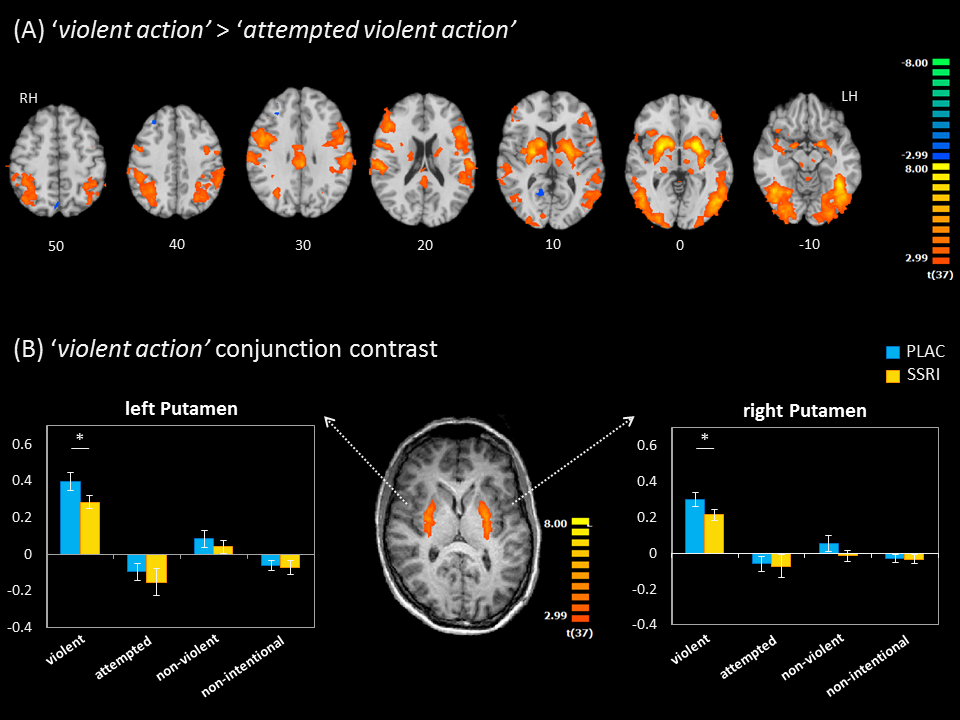

Supplement: Supplementary file 1 — Supplementary material 1 (TIF 265 KB) [file 429_2018_1693_MOESM1_ESM.tif]
