# Supplementary material for: Central serotonin modulates neural responses to virtual violent actions in emotion regulation networks
Source: Brain Struct Funct. 2018 Jun 8;223(7):3327–45. doi: 10.1007/s00429-018-1693-2 (PMC6698268; doi:10.1007/s00429-018-1693-2)
Supplement: Supplementary file 2 — Supplementary material 2 (DOCX 23 KB) [file 429_2018_1693_MOESM2_ESM.docx]

Supplement S1

**Processing reward and reward anticipation during virtual violence**

Results:

We tested the violence-specific effects of the SSRI on the reward system; in particular on the putamen. We contrasts violent actions against the other event types in a conjunction contrast (‘violent action’ > ‘attempted violent action’ n ‘violent action’ > non-violent action’ n ‘violent action’ > non-intended action’). Strong activation clusters emerged in bilateral putamen. For both clusters the averaged beta values entered a two–factor ANOVA differentiating the factors drug (2 level) and event (4 level). The factor *event* yielded significance in both clusters (left: F_1,37_ = 36.14, p < .001; right: F_1,37_ = 24.12, p < .001) and the factor *treatment* failed significance in the right cluster (p > .05), but revealed significance on a trend-level in the left cluster (F_1,37_ = 2.97, p = .09). The interaction *‘drug x event’* failed significance for both clusters. Post-hoc t-tests comparing the placebo with the SSRI condition for each event yielded significance only for the violent action events; the response during violent actions was significantly reduced for the SSRI condition (right: T_38_ = 2.07, p = .045; left: T_38_ = 2.20, p = .034). Notably, the comparison failed significance for attempted violent action as well as for non-violent action and non-intended action.

**Figure caption**

Figure S1: Processing reward and reward anticipation during virtual violence.

(A) The contrast ‘violent action’ vs. ‘attempted violent action’ illustrates reflects confirmed vs. disappointed reward anticipation. Notably, bilateral caudate nucleus (putamen and globus pallidus) are strongly activated. (B) A region-of-interest analysis confirms a violent-specific increased response in bilateral putamen and reveals reduced activation after SSRI exclusively for violent action events. Maps were thresholded according to a corrected p < .05 after Monte Carlo simulation (voxel-wise p < .005 and cluster-size > 8). Post-hoc t-tests: *: p < .05. PLAC: placebo

| Table S1: Reward-related responses during violent actions | | | | | | | |
| --- | --- | --- | --- | --- | --- | --- | --- |
| peak voxel location | cluster size [voxel] | | | peak T-value | peak voxel | | |
|  |  |  |  |  | x | y | z |
| ‘violent action’ > ‘attempted violent action’ (S1A) | |  | |  |  |  |  |
| **frontal cortex:** |  | | |  |  |  |  |
| left inferior frontal | 13458 | | | 6.47 | -41 | -3 | 35 |
| right middle frontal gyrus | 834 | | | -4.91 | 28 | 36 | 35 |
| right inferior frontal gyrus | 367 | | | 4.17 | 55 | 15 | 2 |
|  |  | | |  |  |  |  |
| **parietal cortex:** |  | | |  |  |  |  |
| right postcentral gyrus | 20831 | | | 6.74 | 52 | -24 | 20 |
| left posterior cingulate gyrus | 3850 | | | 7.16 | -2 | -30 | 29 |
| right retroplenial cingulate gyrus | 516 | | | -4.45 | 13 | -54 | 8 |
| right precuneus | 246 | | | -3.81 | 10 | -63 | 44 |
|  |  | | |  |  |  |  |
| **temporal cortex:** |  | | |  |  |  |  |
| left fusiform gyrus | 56020 | | | 8.85 | -41 | -54 | -13 |
| right fusiform gyrus | 35778 | | | 7.78 | 40 | -57 | -7 |
| right superior temporal gyrus | 155 | | | 4.28 | 64 | -6 | 5 |
|  |  | | |  |  |  |  |
| **occipital cortex:** | | |  |  |  |  |  |
| right cuneus | | | 231 | 4.37 | 10 | -84 | 11 |
| left cuneus | | | 139 | 4.39 | -14 | -90 | 20 |
| left lingual gyrus | | | 122 | 3.95 | -5 | -90 | -1 |
|  | | |  |  |  |  |  |
| **subcortex:** | | |  |  |  |  |  |
| right putamen,* | | | 46322 | 10.01 | 13 | 9 | 5 |
| extending into bilateral caudate and globus  pallidus | | |  |  |  |  |  |
| right thalamus | | | 454 | 5.12 | 16 | -27 | 2 |
|  | | |  |  |  |  |  |
| ‘violent action’ conjuction contrast (S1B) | | |  |  |  |  |  |
| right putamen | | | 4156 | 6.27 | -23 | -3 | 8 |
| extending into globus pallidus, caudate and  thalamus | | |  |  |  |  |  |
| left putamen | | | 4057 | 6.35 | 28 | 3 | 8 |
| externding into globus pallidus, caudate and  thalamus | | |  |  |  |  |  |
| right caudate | | | 159 | 6.18 | 10 | 6 | -1 |
| Maps were thresholded at a voxel-wise p < .005 and cluster size k > 8 voxels, corresponding to a p < .05 corrected for multiple comparisons according to Monte Carlo simulations. Cerebellar clusters are not reported. Peak voxel coordinates are given in Talairach space; *: cluster extends to the other hemisphere. | | | | | | | |
